# Indirect evaluation of corneal apoptosis in contact lens wearers by estimation of nitric oxide and antioxidant enzymes in tears

**DOI:** 10.4103/0974-620X.64229

**Published:** 2010

**Authors:** R. P. Bhatia, Shikha Dhawan, H. D. Khanna, Amitabh Dash

**Affiliations:** Department of Ophthalmology, Institute of Medical Sciences, Banaras Hindu University, Varanasi, India; 1 Department of Biophysics, Institute of Medical Sciences, Banaras Hindu University, Varanasi, India; 2 Department of Pharmacology, Institute of Medical Sciences, Banaras Hindu University, Varanasi, India

**Keywords:** Apoptosis, contact lens, glutathione peroxidase, nitric oxide, superoxide dismutase

## Abstract

**Background:**

Contact lens induced trauma to the corneal epithelium results in increased release of inflammatory mediators. The keratocyte apoptosis is directly related to epithelial injury and has been correlated with increased production of nitric oxide. Potent antioxidant enzymes protect cells from oxidative damage by inactivating reactive oxygen species and thus inhibiting apoptosis. This study aims at determination of total nitric oxide and antioxidant enzymes in tears which will be an indirect criteria for assessing apoptosis.

**Materials and Methods:**

Nitric oxide and antioxidant enzymes were estimated in tears of 25 soft contact lens wearers and compared with 25 age and sex matched controls.

**Results:**

Statistically significant increase of nitric oxide (*P*<0.001), superoxide dismutase (*P*<0.001) and glutathione peroxidase (*P*<0.001) levels was seen in tears of contact lens wearers as compared to controls. There was also statistically significant increase in the levels of antioxidant enzymes, superoxide dismutase (*P*<0.05) and glutathione peroxidase (*P*<0.01), with increase in the total duration of contact lens wear in years.

**Conclusions:**

Increase in the level of nitric oxide and antioxidant enzymes in tears of contact lens wearers suggested that contact lens wear suppresses the process of apoptosis. However, it was also postulated that the increased levels of nitric oxide balances the anti-apoptotic activities of increased levels of antioxidant enzymes by its pro-apoptotic activity leading to protective outcomes in contact lens wearers.

## Introduction

Apoptosis is a process of deliberate life relinquishment by a cell in a multicellular organism. It is one of the main types of programmed cell death,[1] and involves an orchestrated series of biochemical events leading to a characteristic cell morphology and death. In contrast to necrosis, which is a form of traumatic cell death that results from acute cellular injury, apoptosis is carried out in an orderly process that generally confers advantages during an organism’s life cycle. The process of apoptosis in controlled by a diverse range of cell signals which may originate either extracellularly or intracellularly.[1] These signals may positively or negatively induce apoptosis.

Mechanical stimulation of corneal surface, due to the physical presence of contact lens, may result in increased release of cytokines, growth factors and other inflammatory mediators.[2] Jalbert and Stapleton[3] and Efron *et al*.[4] showed that keratocyte density in the human cornea decreases during contact lens wear. The contact lens induced keratocyte apoptosis observed by these investigators was attributed to three possible etiologies: hypoxia, cytokine-mediated effects, and mechanically induced effects. Cytokine mediated cytoxicity has been correlated with upregulation of inducible nitric oxide synthase and elevated denovo production of nitric oxide.[5] Nitric oxide has been demonstrated to be involved in the regulation of apoptosis.[6]

Cellular oxidants, called reactive oxygen species (ROS) induce oxidative damage in cells and are essential mediators of apoptosis.[7] Potent antioxidant enzymes such as superoxide dismutase (SOD) and glutathione peroxidase (GSH) protect cells from oxidative damage by inactivating ROS and thus inhibit apoptosis.[8]

This study aims at determination of nitric oxide (NO) and antioxidant enzymes in tears.

- Tear fluid specimen will be taken and subjected to ELISA estimation for determination of levels of NO.
- Tear fluid specimen will be analyzed by spectrophotometric methods for activities of enzymes SOD and GSH.
- Values of NO and antioxidant enzymes in tears will be correlated with the total duration of contact lens wear and daily contact lens wear time.

## Materials and Methods

This study was done at Bhuwalika Eye Hospital, Department of Ophthalmology in conjunction with Department of Biophysics, Institute of Medical Sciences, Banaras Hindu University, Varanasi. The study was reviewed and approved by the Institutional Ethics Committee.

A total of 25 contact lens wearers were included. Out of 25 cases, 20 patients were females and 5 were males and their age ranged from 20 to 30 years. Cases were divided according to total duration of contact lens wear in years and average daily contact lens wear time in hours [Table 1]. All the contact lens wearers were myopic.

**Table 1:** Duration of contact lens wear and average daily contact lens wear time

	*No. of contact lens wearers*	*Percentage*
Duration of lens wear (years)		
≤ 1	6	24
2–4	4	16
5–7	6	24
>7	9	36
Average daily contact lens wear time (hrs)		
<2	4	16
3–6	7	28
7–9	7	28
>9	7	28

### Methods

Tears were collected from controls and contact lens wearers in sitting posture by placing capillary tube at the lateral canthus, without corneal anaesthesia, taking care to ensure that no contact was made with the lid margin and corneal surface. Tears were sucked by capillary action into the tube. Patient was asked to look straight to avoid any trauma. Tears were then immediately transferred to a microtube for the estimation of NO, SOD, GSH.

### Estimation of nitric oxide in tears

The quantitative determination of NO was done using the Assay Designs’ NO assay kit (Assay Designs Inc., USA). The kit involved the enzymatic conversion of nitrate to nitrite, by the enzyme nitrate reductase, followed by the colorimetric detection of nitrite as a colored azo dye product of the Griess reaction that absorbs visible light at 540 nm.

### Estimation of antioxidant enzymes in tears

The activities of two antioxidant enzymes SOD and GSH in tears were analyzed by spectrophotometric methods.

## Results

The mean NO level in contact lens wearers was 127.35 ± 69.58 μmol/L while the mean NO level in controls was 80.90 ± 31.78 μmol/L. The increase in mean NO level in contact lens wearers as compared to controls was statistically significant (*P*<0.001; Table 2).

**Table 2:** Comparison of mean nitric oxide levels, superoxide dismutase level and glutathione peroxidase level in contact lens wearers and controls

	*Contact lens wearers (n=25)*	*Controls (n=25)*	*t value*	*P value*
Nitric oxide levels (μmol/L)	127.35 ± 69.58	80.90 ± 31.78	4.293	<0.001
Superoxide dismutase level (U/ml)	189.43 ± 113.31	65.11 ± 16.40	7.678	<0.001
Glutathione peroxidase level (U/L)	3771.43 ± 2831.79	501.35 ± 383.36	8.092	<0.001

Mean NO level in contact lens wearers did not vary significantly with duration of contact lens wear in years or average daily contact lens wear time in hours (*P*>0.001; Table 3).

**Table 3:** Comparison of mean nitric oxide (μmol/L)levels with duration of wear and average daily contact lens wear time

	*Number*	*Mean ± SD*
Duration of lens wear (years)		
< 1	6	144.07 ± 88.94
2–4	4	118.00 ± 46.48
5-7	6	124 ±69 ± 69.90
>7	9	122.14 ± 67.19
Average daily contact lens wear time (hours)		
<2	4	110.73 ± 66.89
3–6	7	133.43 ± 75.24
7–9	7	122.29 ± 64.79
>9	7	135.84 ± 75.29

The mean SOD level in contact lens wearers was 189.43 ± 113.31 U/ml while the mean SOD level in controls was 80.90 ± 31.78 U/mL. The increase in mean SOD level in contact lens wearers as compared to controls was statistically significant (*P*<0.001) [Table 2].

On comparison of mean SOD level with total duration of contact lens wear in years, the result was statistically significant (*P*<0.05). Mean SOD level did not vary significantly with average daily contact lens wear time in hours (*P*>0.001) [Table 4].

**Table 4:** Comparison of mean SOD levels (U/ml) with duration of wear and average daily contact lens wear time

	*Number*	*Mean ± SD*
Duration of wear (years)		
<1	6	131.99 ± 74.09
2–4	4	135.83 ± 99.16
5–7	6	206.26 ± 129.21
>7	9	240.32 ± 109.45
Average daily contact lens wear time (hours)		
<2	4	123.32 ± 70.18
3–6	7	179.14 ± 108.71
7–9	7	204.38 ± 122.18
>9	7	222.52 ± 121.54

The mean GSH level in contact lens wearers was 3771.43 ± 2831.79 U/L while the mean GSH level in control was 501.35 ± 383.36 U/L. The increase in GSH level in contact lens wearers as compared to controls was statistically significant (*P*<0.001) [Table 2].

On comparison of mean GSH level with total duration of contact lens wear in years, the result was statistically significant (*P*< 0.01). Mean GSH level did not vary significantly with average daily contact lens wear time in hours (*P*>0.001) [Table 5].

**Table 5:** Comparison of mean glutathione peroxidase levels (U/L) with duration of wear (in years) and average daily contact lens wear time (in hours)

	*Number*	*Mean ± SD*
Duration of wear (years)		
<1	6	2261.42 ± 1943.55
2–4	4	2203.94 ± 2201.01
5–7	6	3960.65 ± 2815.27
>7	9	5348.63 ± 2849.82
Average daily contact lens wear time (hours)		
<2	4	2110.36 ± 1944.25
3–6	7	2542.65 ± 2854.81
7–9	7	3847.88 ± 2708.28
>9	7	4872.95 ± 3104.55

## Discussion

Programmed cell death (apoptosis) is the controlled cell death that occurs with minimal collateral damage to surrounding cells or tissue during development, homeostasis and wound healing.[9,10] A cell that undergoes apoptosis, in contrast to necrosis, involutes with little accompanying inflammation and without the release of degradative components that would inflict collateral damage to surrounding tissues.[11] In response to epithelial injury, anterior keratocytes undergo apoptosis. Keratocyte apoptosis is the first change noted after procedures in which the epithelium is injured and might be an initiator of the subsequent wound healing cascade.[12] Responses to mechanical induced injury such as due to contact lens and corneal tissue organization occurs through initiation of keratocyte apotosis.[13]

Contact lens induced trauma to the epithelium results in increased release of cytokines, growth factors and other inflammatory mediators.[2] This cytokines mediated cytotoxicity has been linked with upregulation of inducible NO synthase and elevated denovo production of NO.[5] NO has been shown to be able to induce apoptosis and also to protect from apoptosis in different cell types.[6]

We report the results of an indirect study of apoptosis in contact lens wearers done by estimation of nitric oxide and antioxidant enzymes in tears in contact lens wearers and compared with controls. A statistically significant increase in mean NO level in contact lens wearers as compared to controls was observed. The NO levels did not vary significantly with total duration of lens wear in years and average daily lens wear time in hours. Additionally, there was statistically significant increase in mean SOD level in contact lens wearers as compared to controls. The SOD levels varied significantly with total duration of lens wear in years but did not vary significantly with average daily lens wear time. A statistically significant increase in mean GSH level in contact lens wearers as compared to controls was also detected. The GSH levels also varied significantly with total duration of lens wear in years but did not vary significantly with average daily lens wear time.

Previous studies have shown that ocular tissues and fluids contain high molecular weight antioxidants such as glutathione peroxidase and superoxide dismutase that play a key role in protecting against oxidative damage.[14] Under physiological conditions, these antioxidant enzymes protect the cornea against oxidative stress[15,18] increase in ROS concentration by depletion of antioxidants has been shown to enhance apoptosis. On the contrary, excessive antioxidants decrease ROS level and inhibit apoptosis.[7]

We hypothesize that increased levels of nitric oxide in tears of contact lens wearers possibly supports the apoptotic process. However, a significant increase in the level of antioxidant enzyme activities in tears in contact lens wearers suggests that there is a suppression of the process of apoptosis in contact lens wearers. The former has to nullify the antioxidant enzymes induced anti-apoptotic activity in a favorable way, thereby protecting the corneal and conjuctival cells by apoptosis which is a homeostatic mechanism. However further studies are required to explain fully the effect of changes in the levels of NO and antioxidant emzymes in the tear films of contact lens wearers on corneal apoptosis.
